# SARS-CoV-2 S, M, and E Structural Glycoproteins Differentially Modulate Endoplasmic Reticulum Stress Responses

**DOI:** 10.3390/ijms26031047

**Published:** 2025-01-26

**Authors:** Wejdan Albalawi, Jordan Thomas, Farah Mughal, Aurelia Kotsiri, Kelly J. Roper, Abdullateef Alshehri, Matthew Kelbrick, Georgios Pollakis, William A. Paxton

**Affiliations:** 1 Department of Clinical Infection, Microbiology and Immunology (CIMI), Institute of Infection, Veterinary and Ecological Sciences (IVES), University of Liverpool, Liverpool L69 7BE, UK; waalblawi@ju.edu.sa (W.A.); j.thomas93@live.co.uk (J.T.); farah.mughal@liverpool.ac.uk (F.M.); aurelia.kotsiri@liverpool.ac.uk (A.K.); kelly.roper@apha.gov.uk (K.J.R.); aaalshehre@nu.edu.sa (A.A.); matthew.kelbrick2@liverpool.ac.uk (M.K.); 2 Department Clinical Laboratory Sciences, College of Applied Medical Sciences, University of Aljouf, Sakakah 72388, Saudi Arabia; 3 Virology Department, Animal and Plant Health Agency (APHA-Weybridge), Addlestone KT15 3NB, UK; 4 Department of Clinical Laboratory Sciences, Faculty of Applied Medical Sciences, Najran University, P.O. Box 1988, Najran 61441, Saudi Arabia

**Keywords:** SARS-CoV-2, structural proteins, HIV-1 LTR, endoplasmic reticulum (ER) stress

## Abstract

We have previously shown that the hepatitis C virus (HCV) E1E2 envelope glycoprotein can regulate HIV-1 long-terminal repeat (LTR) activity through disruption to NF-κB activation. This response is associated with upregulation of the endoplasmic reticulum (ER) stress response pathway. Here, we demonstrate that the SARS-CoV-2 S, M, and E but not the N structural protein can perform similar downmodulation of HIV-1 LTR activation, and in a dose-dependent manner, in both HEK293 and lung BEAS-2B cell lines. This effect is highest with the SARS-CoV-2 Wuhan S strain and decreases over time for the subsequent emerging variants of concern (VOC), with Omicron providing the weakest effect. We developed pseudo-typed viral particle (PVP) viral tools that allowed for the generation of cell lines constitutively expressing the four SARS-CoV-2 structural proteins and utilising the VSV-g envelope protein to deliver the integrated gene construct. Differential gene expression analysis (DGEA) was performed on cells expressing S, E, M, or N to determine cell activation status. Gene expression differences were found in a number of interferon-stimulated genes (ISGs), including IF16, IFIT1, IFIT2, and ISG15, as well as for a number of heat shock protein (HSP) genes, including HSPH1, HSPA6, and HSPBP1, with all four SARS-CoV-2 structural proteins. There were also differences observed in expression patterns of transcription factors, with both SP1 and MAVS upregulated in the presence of S, M, and E but not the N protein. Collectively, the results indicate that gene expression patterns associated with ER stress pathways can be activated by SARS-CoV-2 envelope glycoprotein expression. The results suggest the SARS-CoV-2 infection can modulate an array of cell pathways, resulting in disruption to NF-κB signalling, hence providing alterations to multiple physiological responses of SARS-CoV-2-infected cells.

## 1. Introduction

Since the outbreak of SARS-CoV-2, extensive research has concentrated on understanding the molecular consequences of viral infection and its impact on cellular biology. The structural proteins of SARS-CoV-2, including spike (S), membrane (M), envelope (E), and nucleocapsid (N), have become focal points of investigation due to their critical roles in viral replication, assembly, and modulation of host immune responses [1]. The S protein is pivotal for viral entry into host cells [2], binding to the human ACE2 receptor and serving as a primary target for neutralising antibodies [3,4,5]. Positive evolutionary selection has shaped the S protein, resulting in the emergence of SARS-CoV-2 variants with enhanced fitness [6]. Understanding how these mutations in the S protein impact viral function is crucial. As of March 2022, the WHO has identified five SARS-CoV-2 strains as variants of concern (VOC): Alpha, Gamma, Delta, and Omicron (WHO, 2022). Understanding the biological effects of S protein variations will help to guide ongoing research and public health efforts in managing the evolving landscape of the COVID-19 pandemic.

SARS-CoV-2 S, E, and M are structural proteins synthesised, inserted, and folded in the ER before being transported to the ER–Golgi-apparatus intermediate component (ERGIC), where virus particles are assembled, whereas N protein is translated in the cytoplasm [7,8,9,10]. Normal ER function is required for protein synthesis, folding, modification, and transport, whereas ER stress is induced by the accumulation of unfolded/misfolded proteins [11,12]. The unfolded protein response (UPR) is a cryoprotective state induced by ER stress and functions to restore ER homeostasis [13]. To counteract the UPR response, viruses can activate components of the UPR from signals in the ER to promote viral translation and synthesis [14]. This is observed in the synthesis of viral proteins in the ERGIC which activate the ER stress response pathway [15]. It is also commonly observed in avian influenza viruses (AIV), in which the hemagglutinin (HA) glycoprotein undergoes glycosylation modifications in the ERGIC. When there is an accumulation of unfolded proteins in the ER, this activates the UPR and leads to an enhanced virulence, as observed through the activation of cytokine inflammatory levels [16,17]. Other *Betacoronaviruses* such as SARS and human coronavirus HKU1 have been found to enhance UPR-activating activity. The spike proteins from both of these coronaviruses are localised in the ER, inducing ER stress, and have been found to activate the UPR response through activation of the GRP78, GRP94, and CHOP promoters [18]. Similar effects are also observed in flaviruses, adenoviruses, and retroviruses [14].

Several studies have consistently observed elevated serum levels of pro-inflammatory cytokines, including interleukin-1 (IL-1), IL-6, and tumour necrosis factor (TNF), in patients with severe COVID-19 symptoms [19,20,21]. This excessive production of pro-inflammatory cytokines, commonly referred to as a cytokine storm, has been associated with acute respiratory distress syndrome (ARDS) and increased mortality in COVID-19 patients [22]. Similar dysregulated immune responses characterised by hyper-production of pro-inflammatory cytokines have been observed in other viral infections such as H5N1 influenza, SARS-CoV, hantavirus pulmonary syndrome, and possibly in HIV-1 infection, where the absence of negative feedback on the immune response contributes to severe disease outcomes [23,24,25,26]. Viruses have been shown to differentially mediate expression of transposable elements (TEs), which can exert downstream effects by altering host gene expression. In bronchoalveolar lavage from COVID-19 patients, for instance, TEs were significantly overexpressed, while in peripheral blood mononuclear cells, they were downregulated [27].

The nuclear factor κB (NF-κB) signalling pathway, crucial for coordinating the expression of pro-inflammatory cytokines, consists of five key proteins: p65 (RelA), RelB, c-Rel, p105/p50 (NF-κB1), and p100/52 (NF-κB2). These proteins assemble into unique homo- and heterodimeric complexes, which are transcriptionally active due to their interactions [28,29]. This pathway plays a critical role in regulating the immune response, particularly during viral infections [30]. Viruses can exploit the NF-κB pathway for their benefit [31]. In the context of COVID-19, SARS-CoV-2 activates NF-κB, leading to pro-inflammatory cytokine production, which correlates with COVID-19 severity [32]. However, the precise mechanisms by which viral modifications influence NF-κB function remain uncertain. Here, we explore how SARS-CoV-2 structural glycoproteins impact NF-κB activity and modulation of ER stress responses. We utilised the HIV-1 LTR promoter to measure NF-κB activation in response to viral glycoprotein expression and Oxford Nanopore Technologies (ONT) sequencing to identify modulation of ER responses [33,34].

## 2. Results

### 2.1. SARS-CoV-2 Env Glycoproteins Downmodulate HIV-1 LTR Activity

**Figure 1:**
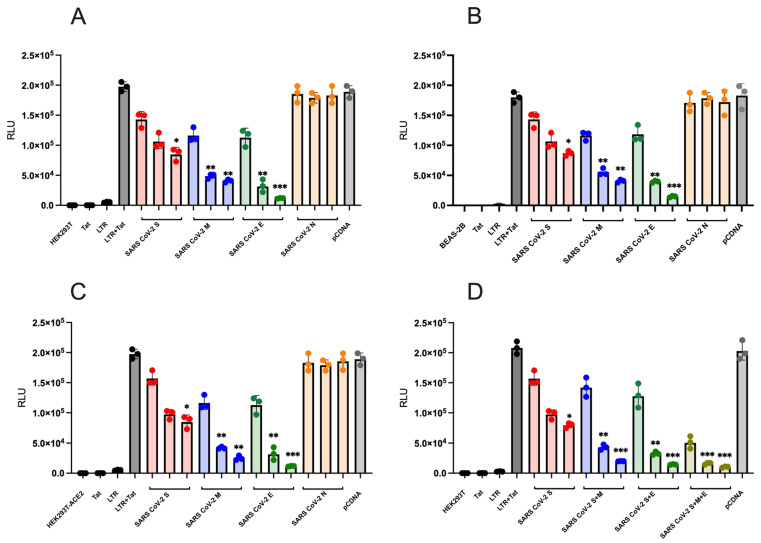
SARS-CoV-2 glycoproteins downmodulate HIV-1 LTR activity. HIV-1 LTR subtype B activation in the presence of varying concentrations of SARS-CoV-2 proteins. (**A**) Effect of SARS-CoV-2 Env glycoproteins on HEK293T cells. (**B**) Effect on BEAS-2B cells. (**C**) Effect on HEK293T ACE2-expressing cells. (**D**) Effect of combined SARS-CoV-2 S, E, and M proteins on HIV-1 LTR activity. Each LTR and Tat was transfected alone as a control for overall LTR activity. Statistical analysis was performed using Kruskal–Wallis and Dunn’s tests to compare the control (LTR + Tat) with each concentration of the SARS-CoV-2 proteins. Significance: * *p* < 0.05, ** *p* < 0.01, *** *p* < 0.001; ns: not significant (*p* > 0.05). The figure illustrates the results of a single experiment, which was conducted in triplicate for validation.

To investigate the impact of SARS-CoV-2 structural proteins (S, M, E, and N) on NF-κB activity, we measured luciferase protein expression driven by the HIV-1 subtype B LTR. Co-transfection experiments were conducted using various concentrations of SARS-CoV-2 protein expression plasmids (12, 6, and 1ng). A fixed amount of reporter LTR plasmid (6ng) expressing the luciferase protein was used in HEK293T (Figure 1A) and BEAS-2B cells (Figure 1B). Co-transfections included a Tat protein expression plasmid alongside the LTR reporter plasmid and SARS-CoV-2 expression plasmids to assess their impact on HIV-1 LTR activation measured by luciferase activity (RLUs). Plasmid quantities were normalised using a pcDNA control plasmid in all experiments, and controls included the transfection of the Tat plasmid alone and the LTR reporter plasmid alone.

Substantial changes in HIV-1 LTR activity were observed in the presence of different SARS-CoV-2 structural proteins. The S protein demonstrated a dose-dependent, inhibitory effect on LTR activation in both cell lines (Figure 1A,B). At the highest concentration (12 ng) of the S protein, a statistically significant suppression of HIV-1 LTR activity was observed (*p* < 0.05). Similarly, the M protein exhibited a dose-dependent modulation of HIV-1 LTR activity, with statistical significance at concentrations of 12 ng and 6 ng (*p* < 0.01) (Figure 1A,B). The E protein significantly downregulated HIV-1 LTR activity in both cell lines, particularly at a concentration of 12ng (*p* < 0.001) (Figure 1A,B). Notably, the inhibitory effect of the E protein surpassed that observed with the S and M proteins. In contrast, the N protein did not affect HIV-1 LTR activity in either cell line tested (Figure 1A,B).

To explore the potential impact of ACE2 receptor expression on SARS-CoV-2 structural proteins’ ability to suppress HIV-1 LTR activity, co-transfection experiments were conducted using HEK293T-ACE2-expressing cells. Results demonstrated that SARS-CoV-2 S, M, and E proteins significantly suppressed HIV-1 LTR activity in HEK293T-ACE2-expressing cells, with no observed effect with the N protein (Figure 1C). These effects were consistent with findings in HEK293T cells (Figure 1A), suggesting that ACE2 receptor expression does not alter the impact of SARS-CoV-2 S, M, and E proteins on HIV-1 LTR activity.

To investigate the combined effect of SARS-CoV-2 M and E proteins with the S protein on HIV-1 LTR activation, HEK293T were co-transfected with variant concentrations of E and M expression plasmids with or without the S protein. Luciferase activity was quantified to analyse the combined effects of SARS-CoV-2 M, E, and S protein expressions on LTR activation. Combining the SARS-CoV-2 S protein with the M or E proteins had a significant effect on HIV-1 LTR activation (*p* < 0.05) with 12 ng and (*p* < 0.05) with 6 ng of plasmid (Figure 1D). Interestingly, combining all SARS-CoV-2 S, M, and E proteins together had an even greater effect on LTR activation, with both 12 ng and plasmid significantly downregulating luciferase activity (*p* < 0.05) (Figure 1D). These results suggest a synergistic effect of combining multiple viral proteins in enhancing the regulatory function of the S protein on LTR transcriptional activity, highlighting the importance of considering the collective impact of viral proteins in modulating NF-κB activity and potentially HIV-1 infection. The S, M, and E proteins exert inhibitory effects on HIV-1 LTR activation, whereas the N protein does not appear to impact HIV-1 LTR activity. This study contributes to our understanding of the molecular interplay between SARS-CoV-2 and HIV-1, offering insights into potential mechanisms underlying the modulation of HIV-1 replication by SARS-CoV-2 structural proteins.

### 2.2. Impact of S Protein Variants on Downmodulation of HIV-1 LTR Activity and Mutation Influence

**Figure 2:**
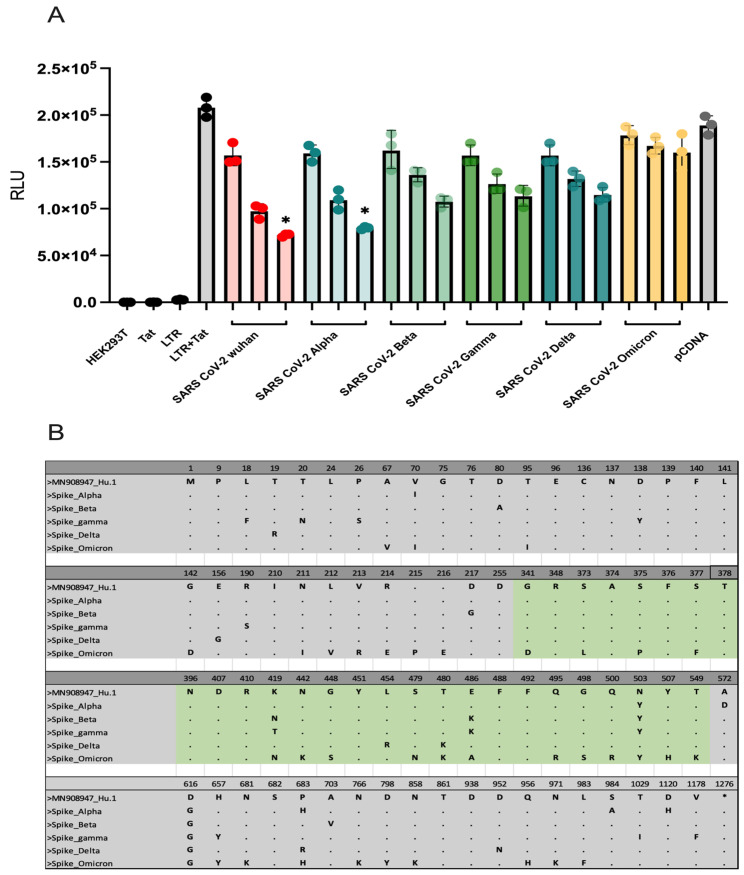
The impact of spike protein mutations on the ability to downmodulate HIV-1 LTR activity. (**A**) Activation of HIV-1 LTR subtype B when co-transfected with varying concentrations of the SARS-CoV-2 Wuhan spike protein, including Alpha, Beta, Gamma, Delta, and Omicron variants. Each LTR and Tat was transfected alone as a control for overall LTR activity. Kruskal–Wallis and Dunn’s tests were used to perform a statistical comparison between the control (LTR + Tat) and each concentration of SARS-CoV-2 spike variant protein. The *p*-value for the concentration of 12ng of SARS-CoV-2 spike and Alpha variants was found to be (* *p* < 0.05). (**B**) Comparative sequence alignment of spike protein amino acid sequences among SARS-CoV-2 wild-type and Alpha, Beta, Delta, Gamma, and Omicron variants. The alignment highlights the positions where variation has occurred in the spike protein when compared to the sequence from the strain (MN908947_Hu.1) first isolated in humans in Wuhan/China. The numbering above indicates the amino acid position within the full-length spike protein, spanning from 1 to 1275. The green highlights indicate the variant amino acid positions within the receptor-binding domain (RBD) region (* *p* < 0.05).

Our initial analysis revealed a concentration-dependent downmodulation of HIV-1 LTR activity by the SARS-CoV-2 S protein, as evidenced by a reduction in luciferase activity at higher concentrations. To delve deeper into the influence of S protein variants on the ability to downmodulate HIV-1 LTR activity, we conducted experiments in HEK293T cells. These cells were transfected with varying concentrations of SARS-CoV-2 plasmids expressing S proteins representing different viral strains (Alpha, Beta, Gamma, Delta, and Omicron), along with LTR luciferase reporter and Tat expression plasmids. Equal plasmid quantities were maintained across all experiments, with pcDNA serving to standardise DNA transfection levels. Our findings indicate that the Alpha variant exerts a comparable effect on downmodulating HIV-1 LTR activity to the SARS-CoV-2 Wuhan S protein, with significant downmodulation observed at a concentration of 12 ng (*p* < 0.05). Conversely, the Beta, Gamma, and Delta variants inhibited HIV-1 LTR activity, although the observed downmodulation did not reach statistical significance (Figure 2A). Intriguingly, the Omicron variant demonstrated no effect on HIV-1 LTR activity. Notably, the Omicron variant harbours a significantly larger number of mutations in the S protein than the other variants (Figure 2B). This observation suggests that SARS-CoV-2 S variants may differ in their capacity to modulate NF-κB signalling, potentially influencing immune induction pathways.

**Figure 3:**
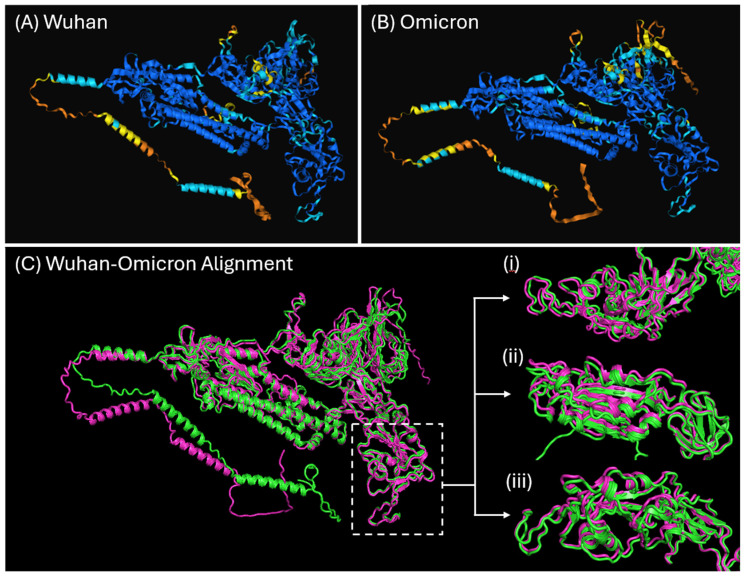
Ribbon representations of AlphaFold predicted structures of the SARS-CoV-2 spike protein of strains: (**A**) Wuhan (pTM = 0.79) and (**B**) Omicron (pTM = 0.81). The residual colour indicates the confidence in the protein structure based on a per-residue measure of local confidence (pLDDT): blue (very high pLDDT > 90), light blue (confident pLDDT 90–70), yellow (low pLDDT 70–50), Orange (very low pLDDT < 50). (**C**) Protein alignment of Wuhan (green) and Omicron (pink), indicating the high similarity between the spike protein structures of the two strains (RMSD = 1.867). The receptor-binding domain (RBD; residue 320–520) is indicated by the dashed rectangle, with further images (C: i, ii, and iii) showing the domain from three angles, highlighting the similarity of the spike protein RBD between the Wuhan and Omicron strains.

Given the number of amino acid alterations within the S structural protein between the Wuhan and Omicron strains, along with the variant effect on modulating LTR activity, we investigated the 3D structure using AlphaFold [35]. The generated protein structures for the Wuhan and Omicron strains were shown to be highly similar (Figure 3A and Figure 3B, respectively). When comparing the more divergent RBD, no discernible differences were observed (Figure 3C), suggesting that our observed results are not structurally determined.

### 2.3. SARS-CoV-2 Differential Gene Expression Analysis

The introduction of SARS-CoV-2 structural proteins (S, M, and E) led to the downregulation of HIV-1 LTR activation. The study aimed to elucidate the mRNA transcriptional profile of cells expressing these proteins, hypothesising an impact on cellular transcription factors like NF-κB. To achieve this, a comprehensive differential gene expression (DGE) analysis was conducted, utilising HEK293T cells stably transduced with SARS-CoV-2 S, M, E, and N proteins, with mock cells as a control group. Three replicates were employed for each condition (mock n = 3, CoV-2 S n = 3, CoV-2 M n = 3, CoV-2 E n = 3, and CoV-2 N n = 3). Total RNA extraction, reverse transcription, and generation of barcoded cDNA libraries through PCR amplification were conducted. Subsequently, the libraries were sequenced on the ONT’s MinION platform, with two libraries per flow cell. R packages, including ‘EdgeR’ and ‘Limma’, were employed for analysis, encompassing barcode and adapter trimming. The sequenced libraries demonstrated consistent characteristics across read count, average read length, and N50 value. Verification of SARS-CoV-2 protein presence in HEK293T cells utilised Kraken2, revealing varying percentages of reads classified as SARS-CoV-2: 0.5% for S and N samples, 2.8% for M samples, and 1% for E samples, while mock samples showed no SARS-CoV-2 reads. Alignment of libraries to the human genome (hg38) and assignment of genomic features facilitated raw gene count generation. DGE analysis involved EdgeR transformation, normalisation using trimmed mean M-values (TMM), and subsequent analysis using Limma. Genes with low expression levels, which may not provide meaningful biological insights, were filtered out using the EdgeR function ‘filterByExpr’ with default parameters. This filtering step resulted in a library that facilitated the comparison of gene expression.

**Figure 4:**
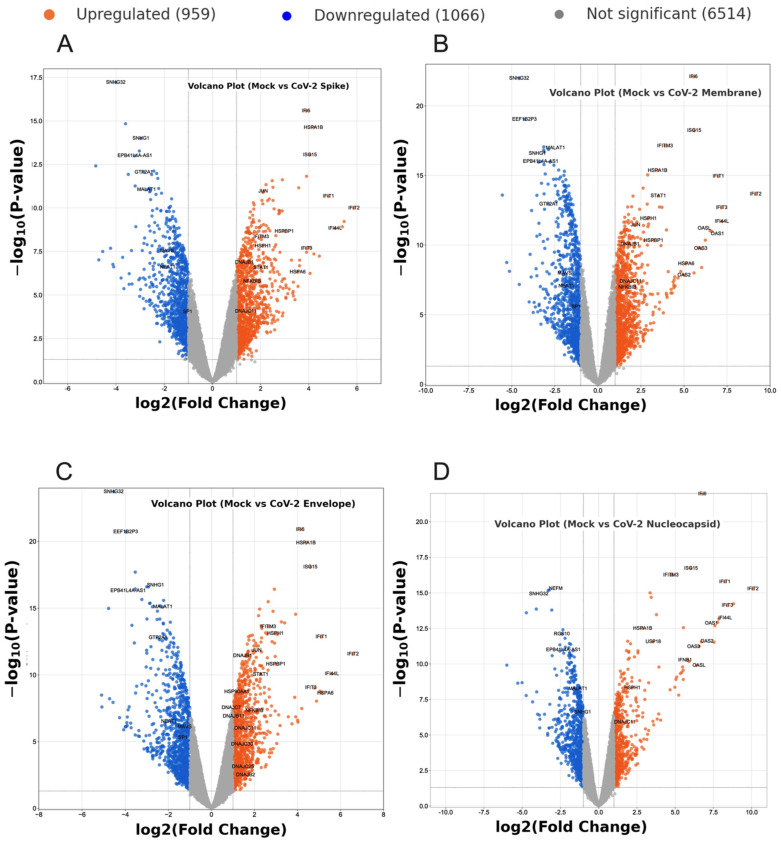
Volcano plots of differential gene expression analysis in SARS-CoV-2 transcriptomics. (**A**–**D**) correspond to specific viral structural proteins: (**A**) spike, (**B**) membrane, (**C**) envelope, and (**D**) nucleocapsid. Volcano plot displaying -Log2 adjusted *p*-value versus Log2 fold change for each gene. Dashed lines indicate the thresholds, with a *p*-value greater than 1.3 -Log10 and a fold change greater than 1 or less than −1. The gene names of the most substantially differentially expressed genes are annotated.

The libraries were normalised using the EdgeR function, applying the trimmed mean M-values (TMM) method. This method determined scaling factors based on the sample with a library size closest to the mean of all samples. The normalised and filtered expression library underwent DGE analysis using the Limma package in R. The Voom function in Limma was initially employed to transform the dataset from counts per million (CPM) to log2 CPM, generating a curve representing the relationship between the square root of the standard deviation of mean log2 CPM and mean expression. This curve demonstrated a decrease in genewise variance with increasing mean expression. Subsequently, Limma was utilised to compare gene expression between mock and SARS-CoV-2 samples using empirical Bayes models. These models effectively reduced the residual variance of genes with high and low variance, enhancing statistical power by shrinking them towards the average. This adjustment ensured that genewise variance was no longer dependent on mean expression levels, providing a robust foundation for the subsequent analyses. The Limma analysis outcomes, including normalised expression levels, log2 fold change (logFC), and *p*-values, were further investigated. Volcano plots illustrated differential gene expression between mock and SARS-CoV-2 S, M, E, and N transduced cells. Significance criteria were set at a log10 adjusted p-value of 1.3 (*p* = 0.05) and a logFC of 1 or −1 (equivalent to a two-fold difference between conditions). Genes meeting both criteria were deemed significantly upregulated or downregulated in the presence of SARS-CoV-2 proteins (Figure 4A–D). According to the volcano plots, numerous genes related to interferon-stimulated genes (ISGs), 2′-5′-oligoadenylate synthetases (OASs), heat shock proteins (HSPs), co-chaperones, and certain transcription factors were significantly upregulated in the presence of SARS-CoV-2 proteins. Conversely, long non-coding RNAs (lncRNAs), transcription factors, and immune-response-related genes were notably downregulated in the presence of SARS-CoV-2 proteins, with specific patterns observed for different structural proteins.

### 2.4. SARS-CoV-2 Proteins Induced Interferon-Stimulated Genes (ISGs)

**Figure 5:**
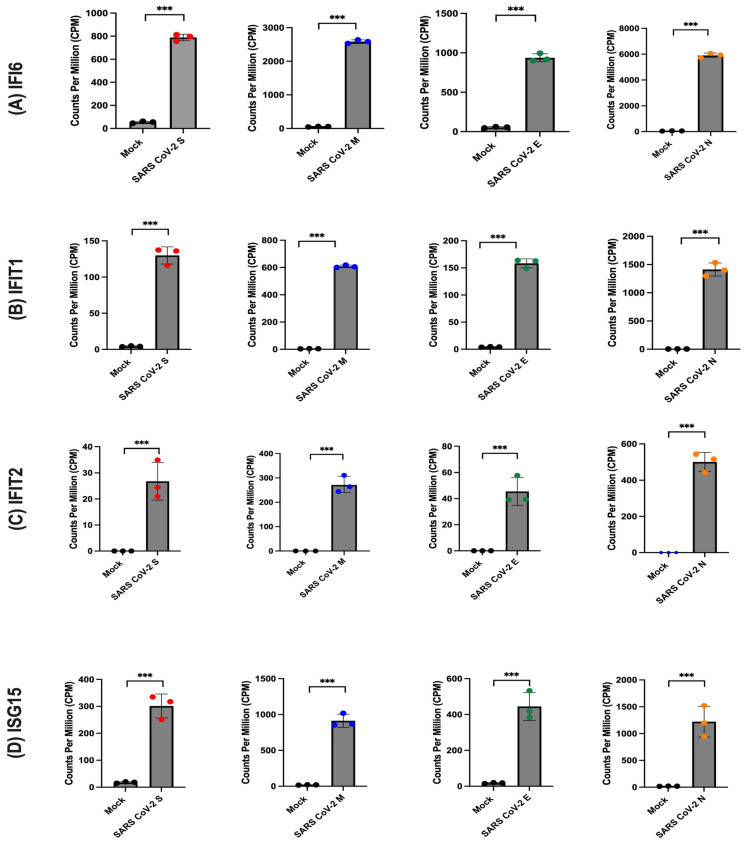
Expression levels of genes associated with interferon-stimulated genes (ISGs) in the presence or absence of SARS-CoV-2 proteins. The normalised counts, expressed as counts per million (CPM), are plotted for each sample group, including mock (n = 3), SARS-CoV-2 S (n = 3), SARS-CoV-2 M (n = 3), SARS-CoV-2 E (n = 3), and SARS-CoV-2 N (n = 3). (**A**) presents the comparison of CPM for IFI16, panel (**B**) for IFIT1, panel (**C**) for IFIT2, (**D**) for ISG15. The statistical significance of differential gene expression was determined using the Voom/Limma analysis, and the results were indicated as ns (not significant), *** *p* < 0.001.

To assess the expression of key genes associated with ISGs across various groups of transduced cells, normalised counts for each gene, expressed as counts per million (CPM), were graphically represented. This analytical approach facilitated a quantitative evaluation of gene expression levels under different experimental conditions. The ensuing results demonstrated a significant increase in the expression of several ISGs, including ISG15, IFI6, IFIT1, IFIT2, IFIT3, IFI44L, and IFITM3, in the presence of SARS-CoV-2 S, M, E, and N proteins, as illustrated in Figure 5. These expression profiles underscore the virus’s capacity to upregulate ISGs. The observed elevation of ISGs, encompassing ISG15, IFI6, IFIT1, IFIT2, IFIT3, IFI44L, and IFITM3, highlights SARS-CoV-2’s ability to activate the host’s interferon response—a critical defence mechanism against viral infection.

### 2.5. SARS-CoV-2 Proteins Induced Heat Shock Proteins (HSPs) and Co-Chaperones

**Figure 6:**
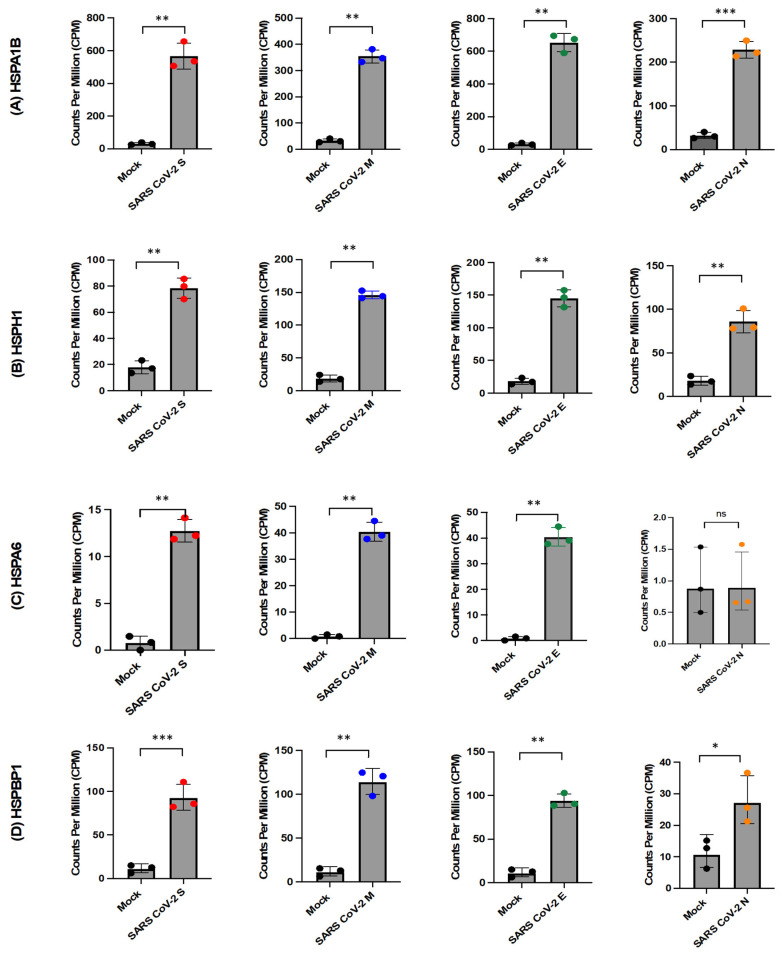
Expression levels of genes associated with heat shock proteins (HSPs) in the presence or absence of SARS-CoV-2 proteins. The normalised counts, expressed as counts per million (CPM), are plotted for each sample group, including mock (n = 3), SARS-CoV-2 S (n = 3), SARS-CoV-2 M (n = 3), SARS-CoV-2 E (n = 3), and SARS-CoV-2 N (n = 3). Panel (**A**) displays the comparison of CPM for HSPA1B, panel (**B**) for HSPH1, panel (**C**) for HSPA6, panel (**D**) for HSPBP1. The statistical significance of differential gene expression was determined using the Voom/Limma analysis, and the results were indicated as ns (not significant), * *p* < 0.05, ** *p* < 0.01, or *** *p* < 0.001.

The analysis of differential gene expression (DGE) revealed that SARS-CoV-2 structural proteins induce significant upregulation of heat shock proteins (HSPs) and co-chaperones. Notably, HSPA6, HSPA1B, HSPBP1, and HSPH1 were identified as HSPs with increased expression. However, the SARS-CoV-2 N protein showed comparatively less stimulation of HSPs compared to other structural proteins. In comparison to mock conditions, the presence of SARS-CoV-2 N protein significantly upregulated only HSPA1B and HSPH1, while HSPBP1 exhibited weaker upregulation with the N protein than with other structural proteins (Figure 6). These findings provide insights into how SARS-CoV-2 interacts with cellular stress response mechanisms through HSPs, suggesting their involvement in cellular stress pathways. The differential stimulation of HSPs by specific viral proteins implies distinct mechanisms by which different SARS-CoV-2 components interact with host cellular machinery.

### 2.6. Expression of SARS-CoV-2 Proteins Regulates Long Non-Coding RNAs (lncRNAs)

**Figure 7:**
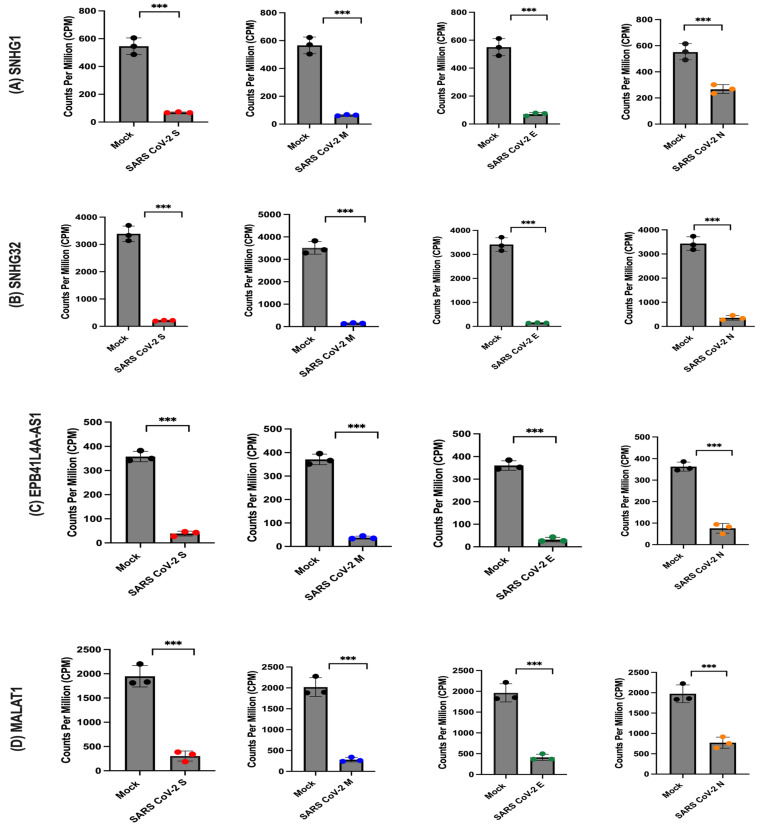
Expression levels of genes associated with long non-coding RNAs (lncRNAs) in the presence or absence of SARS-CoV-2 structural proteins. The normalised counts, expressed as counts per million (CPM), are plotted for each sample group, including mock (n = 3), SARS-CoV-2 S (n = 3), SARS-CoV-2 M (n = 3), SARS-CoV-2 E (n = 3), and SARS-CoV-2 N (n = 3). (**A**) displays the comparison of CPM for SNHG1, (**B**) for SNHG32, (**C**) for EPB4IL4A-AS1, and (**D**) for MALAT1. The statistical significance of differential gene expression was determined using the Voom/Limma analysis, and the results were indicated as ns (not significant), *** *p* < 0.001.

The assessment of gene expression profiles related to long non-coding RNAs (lncRNAs) in cells expressing SARS-CoV-2 proteins involved analysing counts per million (CPM) values for the most significantly different genes. As illustrated in Figure 7, the results indicated a significant decrease in the expression of SNHG1, SNHG32, EPB41L4A-AS1, and MALAT1 in the presence of SARS-CoV-2 proteins. This observation suggests that SARS-CoV-2 proteins negatively influence the expression of these lncRNA-associated genes, revealing a potential regulatory mechanism employed by the virus to modulate host cellular processes through intricate interactions with the transcriptional machinery.

### 2.7. SARS-CoV-2 Proteins Targeting Transcription Factors

**Figure 8:**
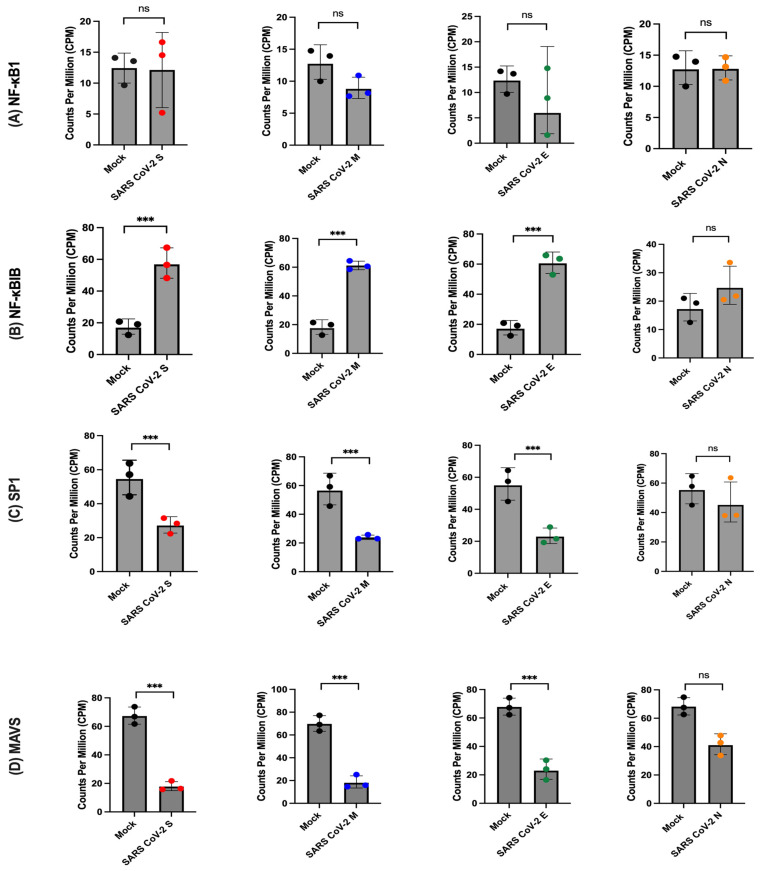
Expression of viral transcription factors in the presence or absence of SARS-CoV-2 proteins. Normalised counts for each gene, expressed as counts per million (CPM), are plotted for each sample group (mock n = 4, SARS-CoV-2 n = 3). (**A**) comparison of CPM for NF-κB1, (**B**) NF-κBIB, (**C**) SP1, and (**D**) MAVS. The statistical significance of differential gene expression was determined using the Voom/Limma analysis, and the results were indicated as ns (not significant), *** *p* < 0.001.

In comparing the transcriptome of cells expressing SARS-CoV-2 structural proteins to a mock condition, DGE analysis revealed alterations in various host transcription factors. We focused on the expression levels of NF-κB1, SP1, JUN, STAT1, and GTF2A1, which were among the most significantly differentially expressed factors (Figure 8). Notably, NF-κB1 did not show a significant difference in expression in the presence of SARS-CoV-2 proteins, but we observed an upregulation of its inhibitor, NF-κBIB (Figure 6A). Furthermore, SP1 and GTF2A1 displayed substantial downregulation in the presence of SARS-CoV-2 S, M, and E proteins, indicating a potential direct influence of these viral proteins on their expression and suggesting an impact on transcription regulation. Conversely, JUN exhibited significant upregulation, implying its involvement in the cellular response to SARS-CoV-2 infection. Additionally, STAT1 showed significant upregulation with all SARS-CoV-2 structural proteins compared to the mock condition, suggesting a role in the host antiviral response by potentially mediating downstream signalling pathways triggered by SARS-CoV-2 infection. Our findings indicate that SARS-CoV-2 proteins have the capacity to modulate the expression of specific transcription factors, potentially influencing host–virus interactions and the host’s antiviral response.

### 2.8. Pathway Enrichment Analysis of SARS-CoV-2 Transcriptome Datasets

**Figure 9:**
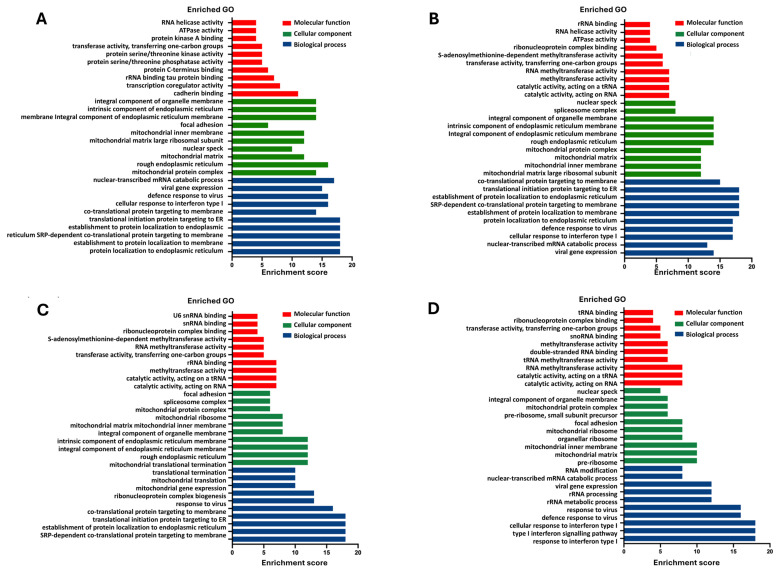
Gene ontology (GO) enrichment analysis for upregulation of DGEs in SARS-CoV-2 spike, membrane, envelope, and nucleocapsid transcriptome. GO annotations show the top 10 terms for significant enrichment of three main categories (biological process, cellular component, and molecular function) with the adjusted *p*-value < 0.05. (**A**) SARS-CoV-2 S, (**B**), SARS-CoV-2 M, (**C**) SARS-CoV-2 E, (**D**) SARS-CoV-2 N.

**Figure 10:**
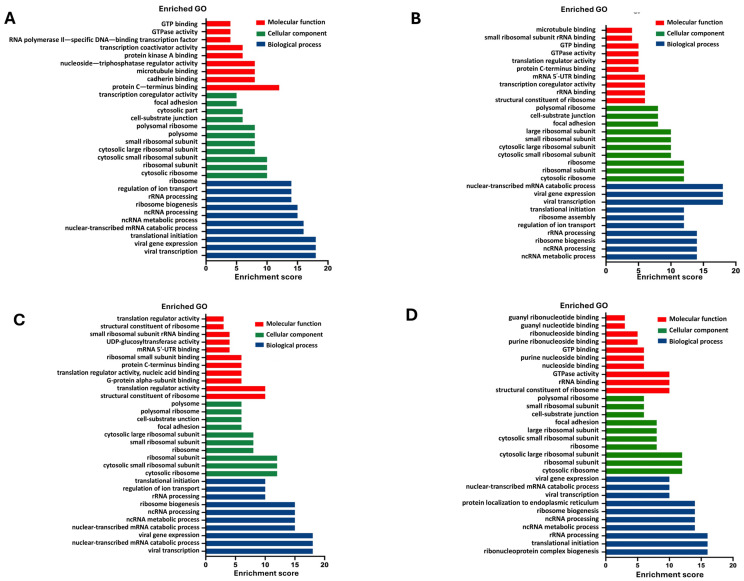
Gene ontology (GO) enrichment analysis for downregulation of DGEs in SARS-CoV-2 spike, membrane, envelope, and nucleocapsid transcriptome. Top 10 terms were plotted for biological process, molecular function, and cellular component in SARS-CoV-2 S (**A**), SARS-CoV-2 M (**B**), SARS-CoV-2 E (**C**), and SARS-CoV-2 N (**D**).

We conducted gene ontology (GO) and gene set enrichment analysis (GSEA) on up/downregulated DGE datasets for SARS-CoV-2 S, M, E, and N transcriptomes compared to control. The analysis encompassed the top 10 enriched terms in biological processes (BP), cellular components (CC), and molecular functions (MF). Focusing on biological processes (BP), upregulated DGEs in SARS-CoV-2 S, M, and E datasets exhibited significant enrichment in processes such as protein localisation to the endoplasmic reticulum (ER) and membrane, co-translational protein targeting, translocation of proteins during translation initiation to the ER, cellular response to interferon type I, defence response to viruses, viral gene expression, and nuclear-transcribed mRNA catabolic process (Figure 9A–C). The SARS-CoV-2 N dataset (Figure 9D) displayed notable differences, as ER-related processes were not significantly enriched. Enriched biological processes in this dataset included the response to interferon type I, defence response to viruses, viral gene expression, rRNA processing, and nuclear-transcribed mRNA catabolic process. Additionally, the downregulated DGEs (Figure 10A–D) shared common BP terms enriched in viral transcription, translational initiation, non-coding RNA metabolism, ribosome biogenesis, rRNA processing, and ion transport regulation across S, M, E, and N datasets.

## 3. Discussion

Several questions regarding SARS-CoV-2 infection and the wide range of symptoms it induces remain unanswered. Notably, the complex interplay between SARS-CoV-2 and the host during infection is incompletely understood. The findings here demonstrate that SARS-CoV-2 S, M, and E Env proteins can downmodulate HIV-1 LTR activity, similar to the observed effects of the HCV E1E2 and sE2 Env proteins [36]. Both SARS-CoV-2 and HCV are positive-sense RNA viruses that replicate in the cytoplasm and have a similar mechanism of viral assembly and release. The fact that SARS-CoV-2 S, M, and E proteins have the same capacity to modulate HIV-1 LTR suggests that this effect could be a result of stimulating the ER stress pathway, which was previously observed in the context of HCV infection. It is well known that the SARS-CoV-2 Env proteins are synthesised, inserted, and folded in the ER [7,8,9], and many studies have suggested that this can induce ER stress responses [37]. The activation of ER stress can result in the upregulation of genes involved in protein folding, degradation, and secretion, which may explain the observed downmodulation of HIV-1 LTR activity induced by SARS-CoV-2 S, M, and E protein expression. The ability of SARS-CoV-2 S, M, and E Env proteins to downmodulate HIV-1 LTR activity may suggest that these proteins play a role in the regulation of host gene expression, potentially contributing to immune evasion and/or pathogenicity. In contrast, the SARS-CoV-2 N Env protein is synthesised in the cytoplasm of infected cells [38,39], unlike the other SARS-CoV-2 structural proteins (S, M, and E). The lack of effect of the N protein on HIV-1 LTR activation likely stems from its cytoplasmic assembly, which limits interaction with ER-associated pathways critical for inducing ER stress and modulating NF-κB signalling. Future studies, such as co-localisation and interaction assays, could further elucidate these findings [8,11].

Here, a separate and comparative transcriptome analysis was conducted on cells stably expressing individual SARS-CoV-2 structural proteins (S, M, E, and N) compared to control cells. Interferons (IFNs) are crucial for the induction of host immune responses against viruses through activation of interferon-stimulated genes (ISGs) [40]. ISGs produce proteins that inhibit viral replication, activate immune cells, and enhance overall host defence mechanisms [40,41]. In the context of SARS-CoV-2 infection, a robust immune response is triggered, leading to the induction of type I interferons (IFNβ and IFNα) and type III interferons (IFNλ) in various cell types [42,43,44,45]. However, despite the induction of interferon responses by related coronaviruses such as SARS-CoV and MERS-CoV, emerging evidence suggests that SARS-CoV-2 may have evolved mechanisms to dampen the production and signalling of type I interferons, allowing the virus to replicate within the host without eliciting an effective antiviral response [46,47]. In a recent study, 23 viral proteins were screened, revealing that SARS-CoV-2 NSP1, NSP3, NSP12, NSP13, NSP14, ORF3, ORF6, and M protein inhibit Sendai-virus-induced IFN-β promoter activation, while NSP2 and S protein have the opposite effect [48].

Through DGEA, we demonstrated that expressing SARS-CoV-2 structural proteins induces the upregulation of specific interferon-stimulated genes (ISGs) such as ISG15, IFI6, IFIT1, IFIT2, IFIT3, IFI44L, IFITM3, and members of the OAS family. These findings indicate that the SARS-CoV-2 proteins have diverse functions in modulating the host’s innate immune response. Similarly, a transcriptomic analysis conducted on SARS-CoV-2-infected calu3 cells demonstrated an upregulation of genes associated with the innate immune response, including IFIT2, OAS2, and IFNB1 [49].

Numerous studies have investigated inflammatory responses induced by coronavirus infection, shedding light on novel roles of heat shock proteins (HSPs) [50,51]. Increased expression of HSPs may play a role in protecting cells from stress induced by the viral infection and aiding in the proper folding and stability of proteins [52]. Similar to the UPR, the HSPs can be upregulated by viruses to aid in replication [50]. HSP genes are central to mitigating ER stress by supporting protein folding and stability, which are critical during viral infections where the unfolded protein response (UPR) is triggered [53]. Suppression of HSPs has been found to lead to lower viral load, such as in porcine epidemic diarrhoea virus infection, where HSP70 knockout inhibited viral replication [54], and in the case of HSPA6 in Enterovirus A71 infection [55], where a reduced viral yield and reduced upregulation of pro-inflammatory cytokines of SARS-CoV-2 were seen in Calu-3 and primary human cells when inhibiting HSP90 [56].

In our study, among the significant upregulated DGEs, many are HSPs encoding genes such as HSPA6, HSPA1B, HSPBP1, and HSPH1. However, the stimulation of HSPs by the SARS-CoV-2 N protein was comparatively lower than that observed with other SARS-CoV-2 structural proteins. In a similar vein, a study investigating the transcriptome of SARS-CoV-2-infected cells revealed substantial upregulation of HSP-encoding genes, including HSPA6 and HSPA1B [57]. These findings support our current study, highlighting the consistent involvement of HSPs and molecular chaperones in the cellular response to SARS-CoV-2 proteins.

Further, we observed significant downregulation of lncRNA-related genes including SNHG1 and SNHG32, EPB41L4A-AS1, MALAT1, and NEAT1 in the presence of SARS-CoV-2 structural proteins. As described above, lncRNAs are emerging as regulatory factors involved in diverse biological processes and have been implicated in the innate immune response, potentially through their interactions with IFN-related pathways [58,59,60]. Emerging evidence suggests that certain long non-coding RNAs (lncRNAs), such as metastasis-associated lung adenocarcinoma transcript 1 (MALAT1) and nuclear paraspeckle assembly transcript 1 (NEAT1), are closely linked to immune responses and may contribute to the inflammatory processes observed in SARS-CoV-2-infected cells [61,62]. Notably, viral-infection-induced downregulation of MALAT1 expression has been associated with enhanced activation of IRF3 and production of type I IFNs, suggesting its antiviral role in therapy [63]. However, our current understanding of the specific functions of lncRNAs in innate immune regulation related to interferons in COVID-19 patients is limited.

Additionally, we observed alterations in the expression of various transcription factors upon exposure to SARS-CoV-2 structural proteins. Specifically, we observed upregulation of JUN and STAT1, while NF-κB1 remained unchanged. Interestingly, the inhibitor of NF-κB, NFκBIB, was shown to be upregulated. Additionally, we observed downregulation of SP1 and GTF2A1. Notably, the influence of the N protein was observed only on the upregulation of STAT1. These findings suggest that SARS-CoV-2 structural proteins have selective effects on the expression of transcription factors, potentially impacting the transcriptional regulation of target genes involved in immune responses and other biological processes. It is worth mentioning that in the presence of S, M, and E proteins of SARS-CoV-2, MAVS, which is a key adaptor protein in the innate immune response and a critical component in the activation of the type I interferon pathway, was downregulated. MAVS is responsible for sensing viral components and initiating downstream signalling that leads to interferon production [63]. The downregulation of MAVS is likely to affect NF-κB signalling, as MAVS is involved in the activation of NF-κB during viral infection [63,64]. Consequently, the decreased MAVS expression may result in impaired NF-κB activation. Similarly, SP1, a transcription factor involved in the regulation of various genes, including those associated with immune responses, was also downregulated. Despite the downregulation of MAVS and SP1, the observed upregulation of interferon in the transcriptome data suggests the involvement of compensatory or alternative mechanisms in the regulation of interferon response during SARS-CoV-2 protein transfection.

Based on the GO analysis, a significant enrichment of upregulated genes in the presence of SARS-CoV-2 proteins was observed in biological processes. Specifically, these upregulated genes were found to be enriched in ER-related processes, cellular response to interferon type I, and defence response to viruses. Reactome pathway analysis further supported these findings, revealing similar enriched pathways among the upregulated genes in the presence of SARS-CoV-2 proteins. These pathways included cytokine signalling in the immune system, interferon alpha and beta signalling, antiviral mechanisms stimulated by interferons, unfolded protein response, regulation of HSF1-mediated heat shock response, and activation of IRE1-alpha and chaperones. This finding is consistent with a study by Sun et al. investigating the transcriptome of SARS-CoV-2-infected cells [58]. The SARS-CoV-2 S, M, and E protein datasets exhibited similar patterns of pathway activation, suggesting a common impact on cellular processes. However, the SARS-CoV-2 N protein dataset displayed distinct differences, with the activation of additional pathways involved in immune signalling, such as TRAF6-mediated IRF7 activation and TRAF6-mediated NF-κB activation. Interestingly, pathways associated with ER stress did not show significant enrichment in the N protein dataset.

There have been several studies reporting dysregulation of ion transport in SARS-CoV-2-infected cells [65,66,67], which aligns with our findings of downregulated ion transport pathways in the presence of SARS-CoV-2 proteins. The downregulation of ion transport observed in our transcriptome data, along with the activation of ER stress pathways, is consistent with previous studies that have reported decreased mRNA and protein levels of Na,K-ATPase subunits in SARS-CoV-2-infected cells and postmortem lung tissue samples from COVID-19 patients [65,66]. These findings suggest a potential association between SARS-CoV-2 infection, ER stress, and impaired ion transport. The impairment of Na,K-ATPase maturation and its delivery to the cell membrane, resulting from disrupted chaperone-assisted folding in the ER lumen, as well as the possible interference of the SARS-CoV-2 spike protein with glycosylation-dependent protein folding machinery, may contribute to the observed decrease in Na,K-ATPase abundance at the plasma membrane [65,66]. Together, these findings highlight the intricate interplay between viral infection, cellular processes, and dysregulation of ion transport, providing valuable insights into the pathogenesis of COVID-19.

## 4. Materials and Methods

### 4.1. Cell Culture

This study used the following human cell lines: the human transfection-competent embryonic kidney cell line, HEK293T, and the human bronchial epithelial cell line, BEAS-2B. All cells were cultured in Dulbecco’s modified Eagle medium (DMEM, Thermo Fischer Scientific, Waltham, MA, USA), supplemented with 10% heat-inactivated foetal bovine serum (ΔFBS, Thermo Fischer Scientific, Waltham, MA, USA), 2 mM/mL L-glutamine (Thermo Fischer Scientific, Waltham, MA, USA), and 1% penicillin–streptomycin (Pen–Strep), (Thermo Fischer Scientific, Waltham, MA, USA). Incubation was carried out at 37 °C with 5% CO_2_.

### 4.2. Plasmid Preparations

Plasmid preparations were conducted through heat shock transformation, followed by DNA purification using Qiagen maxiprep kits (Qiagen, Hilden, Germany). Initially, 2 µL of plasmid were added to One Shot TOP10 chemically competent *Escherichia coli* (Thermo Fischer Scientific, Waltham, MA, USA), followed by a 30 min incubation on ice, 30 s incubation at 42 °C, and a further 2 min incubation on ice. After adding 0.25 mL SOC media, the mixture was incubated at 225 rpm for 1h at 37 °C. The culture was plated on antibiotic-selective agar plates, with resultant single colonies being selected and grown in brain–heart infusion (BHI) broth with ampicillin (100 µg/mL) for miniprep isolation and subsequently used for maxiprep extraction.

### 4.3. Transfection of Cell Lines with LTR and SARS-CoV-2 Protein Plasmid Constructs

The SARS-CoV-2 (Wuhan-Hu-1) S, M, E, and N genes were cloned into the pCDNA3.1 expression plasmid (produced by GeneArt Gene Synthesis). This plasmid was also utilised for cloning SARS-CoV-2 variants (produced by GeneArt Gene Synthesis). HEK293T and BEAS-2B cell lines were seeded in 96-well plates at a density of 1.5 × 10^4^ cells per well (100 µL DMEM) and incubated overnight. The medium was then replaced with 50 µL of Opti-MEM. Transfection mixes were prepared by combining 1 ng of Tat plasmid, 6 ng of LTR-luc plasmid, and 12, 6, or 1 ng of SARS-CoV-2 S, M, E, and N SARS-CoV-2 variants [66], or pCDNA plasmid with 2 µL of Opti-MEM. An additional 2 µL of polyethylenimine (PEI) (0.14 μg/μL) was added, and the mixture was incubated at room temperature for 30 min. The resulting transfection mixture was added to the cells, which were then incubated for 6 h, followed by a 48 h incubation in DMEM. Luciferase activity measurement was conducted after this period.

### 4.4. Stable Cell Line Production

The SARS-CoV-2 (Wuhan-Hu-1) S, M, E, and N genes were integrated into a lentiviral expression plasmid (pGenlenti vector) from Genscript, (Genscript, Piscataway, NJ, USA). Lentivirus particles were generated using pseudo-typed viral particles (PVP) with vesicular stomatitis glycoprotein (VSV-g) for enhanced stability and broader tropism. A transient three-plasmid transfection method was employed, with 3 × 10^6^ HEK293T (lentiX) cells seeded one day before transfection. The lentiviral construct (4500 ng), lentiviral backbone (p8.91, 3000 ng), and VSGg plasmid (2700 ng) [68] were co-transfected using PEI in OptiMEM (Thermo Fischer Scientific, Waltham, MA, USA). After a 48h transfection, lentivirus particles were harvested, filtered (0.45 µM), and stored at −80 °C. Antibiotic selection was optimised using a puromycin kill curve on HEK293T cells (1 × 10^6^). Cells were seeded in two 10 cm^2^ plates in complete DMEM media. After polybrene treatment (8 μg/mL) for 2 min, one plate received lentiviral supernatant, while the other served as a control. After two rounds of transduction and a 48 h puromycin treatment (1 μg/mL), media/antibiotic were refreshed every two days until all control cells were dead. Western blot analysis assessed the expression of the gene of interest.

### 4.5. Flow Cytometry for Transduction Validation

To confirm SARS-CoV-2 protein expression in transduced cells, lentiviral vectors expressing the GFP reporter protein were generated. Lentivirus vector production, infection, and antibiotic selection followed previous protocols. Approximately 1 × 10^5^ cells were used for GFP signal quantification. After pelleting and washing with FACS buffer, cells were resuspended in 200 μL FACS buffer and analysed using flow cytometry. Flow cytometry data were analysed with FlowJo version 7.10.0.

### 4.6. Validation of SARS-CoV-2 Stable Cells

Western blotting was employed to assess the expression levels of SARS-CoV-2 S, N, E, and N proteins in the transduced cells. Cells (1 × 10^6^) were washed with ice-cold dPBS and lysed using radioimmunoprecipitation assay (RIPA) buffer supplemented with a 1% protease inhibitor cocktail. The protein concentration was determined using the Bradford assay with the protein assay kit. Subsequently, 50 µg of cell lysates were mixed with 4× NuPage LDS Sample Buffer and 10× NuPage reducing agent, incubated at 72 °C for 10 min, and separated using polyacrylamide gels (NuPAGE 12% Bis-Tris Gels (Thermo Fischer Scientific, Waltham, MA, USA) via electrophoresis at 120 V for 60 min. The separated proteins were transferred to iBlot™ 2 PVDF Mini Stacks membranes using the iBlot 2 Dry Blotting system. The membranes were blocked using the iBind solution kit and incubated with primary and secondary antibodies for 3 h using the iBind device. Antibodies included rabbit anti-SARS-CoV-2 S 1:500 (SinoBilogical, Beijing, China), rabbit anti-SARS-CoV-2 N 1:500 (Thermo Fischer Scientific, Waltham, MA, USA)), rabbit anti-SARS-CoV-2 M protein 1:250 (Thermo Fisher, Scientific, Waltham, MA, USA)), rabbit anti-SARS-CoV-2 E protein 1:250 (Thermo Fisher Scientific, Waltham, MA, USA), rabbit anti-β-actin 1:250 (Abcam, Cambridge, UK) and horseradish peroxidase (HRP)-conjugated secondary antibody (anti-rabbit IgG) 1:1000 (Thermo Fischer Scientific, Waltham, MA, USA). Protein bands were visualised using the Pierce™ ECL Plus Western Blotting Substrate.

### 4.7. SARS-CoV-2 Transcriptomics

Stable cells expressing SARS-CoV-2 proteins were plated at 4.8 × 10^5^ cells/well in a six-well plate and incubated for 48 h. After replacing the media with 50 μL Opti-MEM, cells were rinsed with warm dPBS and lysed using 350 μL Qiagen buffer RLT with β-mercaptoethanol. RNA purification was performed from cellular lysates using the Qiagen RNAeasy Plus kit, following the manufacturer’s instructions, and subsequently eluted in 35 µL nuclease-free water. Quantification of RNA was carried out using a Qubit high-sensitivity RNA fluorometer. PolyA+ mRNA was purified from 35 ng total RNA using the Dynabeads mRNA purification kit (Thermo Fischer Scientific, Waltham, MA, USA) and eluted in 15 µL of nuclease-free water. Library preparation involved 30 ng polyA+ mRNA and followed the Oxford Nanopore (Oxford, UK) SQK-PCS-109 protocol, incorporating the EXP-PBC-001 barcoding kit for flow cell multiplexing. In brief, reverse transcription was conducted using Maxima H Minus Reverse Transcriptase (42 °C for 90 min, 85 °C for 5 min) (Thermo Fischer Scientific, Waltham, MA, USA). The resulting reverse transcription product (5 µL) was divided into two 50 µL reactions with Oxford Nanopore barcoded primers for amplification (95 °C for 5 min and 12 cycles of 95 °C for 15 s, 62 °C for 15 s, and 65 °C for 8 min, with final elongation at 65 °C for 8 min). Amplification products were pooled and purified using Beckman Coulter Ampure XP magnetic beads (Beckman Coulter, Brea, CA, USA) with a 0.45× ratio of beads to DNA volume. Library quantity assessment was conducted using Qubit, and each library (150 ng) was sequenced with two samples per flow cell. Sequencing was performed using MIN-106 flow cells (R9.4.1 pores) on the Oxford Nanopore MinION sequencing platform (Oxford, UK). Run durations ranged from 24 to 48 h.

### 4.8. Bioinformatics

Guppy (Oxford, UK) base-caller, configured with a minimum score of 7 and fixed parameters for q-score filtering and barcode kits, was employed to base-call and demultiplex the Fast5 files. An additional parameter was introduced to trim primers and adapters from the sequences. To confirm the expression of the protein of interest, Kraken2 (Baltimore, Maryland, USA) mapped the reads to a viral genome database [69], while Minimap2 (Cambridge, UK) established an index from the Genome Reference Consortium Human Build 38 (hg38). Minimap2 was further utilised to map the reads to the indexed hg38 [70], and the ‘Subread’ package version within R (version 4.3.0) (Vienna, Austria)was used for their assignment to genomic features. Subsequently, EdgeR (version 3.42.4), transforming raw counts into counts per million (CPM) and applying additional normalisation using trimmed mean M-values (TMM), was employed to normalise raw read counts. The ‘Voom’ method within Limma (version 3.56.2) was used to fit linear models, identifying differentially expressed genes in the resulting expression file [71,72]. Finally, the normalised count file underwent analysis with the clusterProfiler R package (version 4.8.3) [73] for gene set enrichment analysis, incorporating Gene Ontology (GO) terms to categorise genes based on biological processes, molecular functions, and cellular components. Additionally, the GSEA software (version 4.2.3) (San Diego, California, USA) [74] was employed to assess the enrichment of predefined gene sets. Pathway visualisation utilised the Pathview R package [75], generating pathway diagrams illustrating affected genes and their interactions within specific biological pathways. Reactome and KEGG databases (Kyoto, Japan) served as sources of gene sets for the GSEA analysis.

### 4.9. Protein Structure Prediction

The SARS-CoV-2 Wuhan and Omicron spike protein structures were modelled using AlphaFold3 (Cambridge, UK), providing predicted template modelling (pTM) and a per-residue measure of local confidence (pLDDT) metric indicating model accuracy [35]. Protein alignments and root mean square deviation (RMSD) calculations were performed using PyMOL version 3.0 (Schrödinger, New York, NY, USA) [76].

### 4.10. Statistical Analysis

Statistical analyses were conducted using GraphPad Prism version 9.0 (Boston, Massachusetts, USA) for windows. Unpaired sample comparisons were performed for all data. Non-parametric one-way analysis of variance (ANOVA) using the Kruskal–Wallis test was applied, followed by Dunn’s analysis for paired multiple comparisons. Data are presented as mean ± SEM, with error bars representing the SEM. A significance level of *p* < 0.05 was considered appropriate. Further details about the statistical methods employed can be found in the corresponding figure legend.

## 5. Conclusions

Comparative analysis of individual SARS-CoV-2 structural proteins revealed compelling insights into the molecular mechanisms driving COVID-19 pathogenesis. The findings demonstrate the potential impact of these viral proteins on host immune responses, transcriptional regulation, and cellular stress pathways. We observed robust upregulation of interferon-stimulated genes (ISGs) and activation of antiviral defence pathways. The presence of SARS-CoV-2 structural proteins induced the upregulation of ISGs, highlighting their role in modulating host immune defences. Additionally, the activation of HSPs and the UPR suggested the imposition of ER stress by SARS-CoV-2 proteins. Notably, the N protein exhibited distinct regulatory properties, with less stimulation of HSPs compared to other viral proteins. This finding supports the finding that the downmodulation of HIV-1 LTR activation was associated with the upregulation of ER stress responses.

Dysregulation of transcription factors indicates their involvement in the cellular response to SARS-CoV-2 infection. The differential effects of the N protein on transcription factors highlight its unique role in modulating gene expression patterns. These findings underscore the complexity of SARS-CoV-2–host interactions and the need for further research to understand the underlying molecular mechanisms. Advancing our understanding of these interactions will be crucial for developing targeted therapeutic approaches for COVID-19. In summary, our study provides compelling evidence of the intricate interplay between SARS-CoV-2 structural proteins, host immune responses, transcriptional regulation, and cellular stress pathways, shedding light on COVID-19 pathogenesis.

## Data Availability

The authors welcome requests for access to the data generated in this study, which will be made available by the corresponding authors.
